# Neural correlates of proactive and reactive motor response inhibition of gambling stimuli in frequent gamblers

**DOI:** 10.1038/s41598-017-07786-5

**Published:** 2017-08-07

**Authors:** D. Brevers, Q. He, B. Keller, X. Noël, A. Bechara

**Affiliations:** 1 Department of Psychology, and Brain and Creativity Institute, University of Southern California, Los Angeles, CA USA; 2 Psychological Medicine laboratory, Faculty of Medicine, Brugmann-campus, Université Libre de Bruxelles, Brussels, Belgium; 3 Faculty of Psychology, Southwest University, 2 Tiansheng Rd, Chongqing, China

## Abstract

We used functional magnetic resonance imaging to examine whether motivational-salient cues could exert a differential impact on proactive (the restrain of actions in preparation for stopping) and reactive (outright stopping) inhibition. Fourteen high-frequency poker players, and 14 matched non-gambler controls, performed a modified version of the stop-signal paradigm, which required participants to inhibit categorization of poker or neutral pictures. The probability that a stop-signal occurs (0%, 17%, 25%, 33%) was manipulated across blocks of trials, as indicated by the color of the computer screen. Behavioral analyses revealed that poker players were faster than controls in categorizing pictures across all levels of proactive motor response inhibition (go trials). Brain imaging analyses highlighted higher dorsal anterior cingulate cortex activation in poker players, as compared to controls, during reactive inhibition. These findings suggest that, due to their faster rates of stimulus discrimination, poker players might have recruited more cognitive resources than controls when required to stop their response (reactive inhibition). Nevertheless, no main effect of stimulus type was found, on either proactive or reactive inhibition. Additional studies are, therefore, needed in order to confirm that investigating the dynamics between reactive and proactive inhibition offers a discriminative analysis of inhibitory control toward motivational-salient cues.

## Introduction

Gambling has never been so easily accessible and readily available. It is possible to bet everywhere, at every moment, and simultaneously using different platforms. The high volume of gambling advertising is also there to remind you to do so. As a matter of fact, gambling cues are present in newspapers, TV, radio, sports arenas, and the Internet^1–3^. A main consequence of this increased exposure is that it could foster temptation to gamble in frequent gamblers^4^.

Congruent with this view, functional magnetic resonance imaging (fMRI) studies highlighted that problem gamblers exhibit increased activation in salience/motivational brain circuitry while viewing gambling pictures (refs 5 and 6, but also see ref. 7). Neural activations include areas involved in emotional processing (amygdala^8^), reward anticipation (ventral striatum^9^), and affective decision-making (ventrolateral prefrontal cortex, VLPFC^10^). Interestingly, gambling cue reactivity was also associated with anterior cingulate cortex (ACC) and dorsolateral prefrontal cortex (DLPFC) activations^5, 6^. Brain activations within these areas overlap with some of the neural pathways underlining motor response inhibition, namely the ACC and the DLPFC, which are often involved in the anticipation/preparation of response conflicts, and in selecting superordinate sets of action-selection rules^11^. It follows that enhanced resources engaged in the processing of salient stimulus should leave fewer resources available for effortful control^12–14^. Hence, one may advance the notion that motivational salience directed at gambling cues could lower gamblers’ ability to inhibit a motor response toward those stimuli.

Currently, only van Holst and colleagues^15^ have examined the interaction between stimulus saliency and motor response inhibition in gamblers. Consistent with previous work on cue reactivity, this fMRI study highlighted the idea that gambling-related stimuli are flagged as more salient by individuals with problem gambling. This was reflected by higher DLPFC, ACC and ventral striatal activations, as compared to non-gambler controls. Surprisingly, problem gamblers were also better than controls at inhibiting their motor response toward gambling cues, and showed lower activation of the DLPFC and ACC regions. One explanation for this result is that this sample of gamblers was recruited from addiction treatment centers, where they received cognitive behavioral therapy. This could have lowered their motivational-approach tendencies towards gambling cues, i.e., stimuli that are related to their abstinence/moderation goals^16–19^. Accordingly, enhanced response inhibition toward food cues has been evidenced in overweight adults who are high on dietary restrain^20–22^. By contrast, research has shown that motivationally salient stimuli (e.g., erotica, food, alcohol, drugs, tobacco, gambling) could hamper motor response inhibition in both frequent and (non-restricting) problem users^23, 24^. Besides, in van Holst *et al*.^15^, the type of stimuli was not adapted to gamblers’ preferred type of gambling (e.g. poker vs. blackjack). This could have further decreased the motivational-salience attached to gambling cues, and its subsequent (detrimental) impact on motor response inhibition. For instance, Pessoa and colleagues^25^ showed that low-emotional stimuli increased motor response inhibition, and the opposite was found for high-emotional cues. Thus, motor response inhibition should be decreased in (non-restricting) frequent gamblers who are required to inhibit gambling cues that correspond to their daily-life gambling habits.

Another gap of knowledge from the literature is whether salient-motivational cues interfere with both *proactive* and *reactive* motor response inhibition, which refer to distinct temporal dynamic modes of motor response inhibition^26–28^. Proactive inhibition can be conceptualized as a form of “early selection” in which goal-relevant information is actively maintained to optimally bias attention, perception and action systems in a goal-driven manner, and is used to restrain actions in preparation for stopping (e.g. slowing down while driving along a school). Reactive inhibition is a “late correction” process, which is triggered by external signals (e.g. to brake when a pedestrian suddenly cross the street), and results in the stopping of the ongoing action. Put differently, under reactive control, goal representations are only activated at the time in which they are needed^26, 28^. Proactive control, by contrast, relies upon the anticipation and prevention of the stop signal before it occurs^26–28^. At a neural level, it has been demonstrated that proactive and reactive inhibition activate common brain areas, including the superior, middle, and inferior frontal gyrus, supplementary motor area, angular gyrus, and the striatum, in both right and left hemispheres^29–40^. This shared neural pathway suggests that proactive and reactive inhibition might rely on a common network involved in “braking” motor output when it is weakly activated (proactive inhibition), and in “stopping” motor output completely when strongly activated (reactive inhibition)^26, 36^. Proactive and reactive inhibition might also activate specific neural pathways, with reactive inhibition associated with right DLPFC, right VLPFC and anterior supplementary motor area activations, which could reflect stimulus-driven attention, and action reprogramming from going to stopping^36, 39, 41–43^. Proactive motor control has been reported to specifically activate the right and left superior parietal lobule, which could reflect top-down influence over motor control^36, 39^.

The importance of examining both proactive and reactive components of motor response inhibition has been further emphasized by recent fMRI studies undertaken in sub-clinical and clinical samples^33, 38^. Zandbelt and colleagues^38^ showed that, in comparison to control subjects, proactive inhibition was reduced in schizophrenia patients, while reactive inhibition did not differ between the two groups. More specifically, as compared to controls, patients failed to slow down responding as the probability that they may have to stop increased (i.e., reduced proactive inhibition). At a brain imaging level, this pattern was associated with a failure to activate the right striatum, the right inferior frontal cortex, and the left and right temporoparietal junction. In another study, van Rooij and collaborators^33^ highlighted that war veterans with posttraumatic stress disorder (PTSD) showed reduced reactive inhibition (i.e., slower stop-signal reaction time, SSRT), as compared to non-military controls, along with a decreased inhibition of the left pre/postcentral gyrus. The veterans with PTSD also exhibited impaired behavioral proactive inhibition. Furthermore, the PTSD group showed a reduced right inferior frontal gyrus response during proactive inhibition, as compared to a group of veterans without PTSD. Taken together, these findings confirm that investigating both reactive and proactive inhibition might offer a more nuanced, discriminative and fine-grained analysis of inhibitory control.

The goal of this study was to examine, at both behavioral and neural levels, the impact of gambling cues on proactive and reactive motor response inhibition in high-frequent gamblers. Based on the aforementioned argumentation, we recruited one specific “type” of gamblers (17 high-frequent poker gamblers; sample size based on van Holst *et al*.^15^) to inhibit cues that matched their preferred gambling games (poker pictures). At a behavioral level, we predicted that, when compared to a group of non-gambler controls (*n* = 16), poker players would exhibit lower proactive and reactive inhibition while categorizing poker cues. Second, we expected that poker players would exhibit lower brain activation than controls during proactive and reactive inhibition. Third, in line with previous neuroimaging studies on gambling cue reactivity^5, 6, 15^, we also hypothesized that poker players, as compared to controls, would show enhanced brain activity during the viewing of poker pictures, especially in the salience/motivational brain circuitry (e.g., amygdala, striatum, VLPFC).

## Results

### Behavioral task validation

The SST was first pretested in a sample of students (*N* = 16). Those pretests aimed to test whether categorization RT is modulated by the level of stop-signal probability (green < yellow < orange < red). In addition, we expected that (i) mean *p*[respond|signal]) (pooled across the yellow, orange, and red contexts) will approximate 0.50, which would confirm the effectiveness of the tracking procedure; and (ii) mean failed stop-signal RT should be faster than mean go signal RT (both measures pooled across the yellow, orange, and red contexts), which would be used as a criterion of independence between the finish times of the *go* and the *stop* responses (for further details on data analyses, see Supplementary Materials).

These analyses revealed that: categorization RT (in ms) increased as a function of the level of stop-signal probability (*F*(3,15) = 100.51, *p* < 0.0001, *η* ^*2*^ = 0.87; see also Figure S1 in Supplementary Materials); that mean *p*[respond|signal]) was close to 0.50 for both the neutral (M = 0.49, SD = 0.04) and the poker (M = 0.48, SD = 0.04) stimuli; and that the mean failed stop-signal RT (M = 857, SD = 87) was lower than the mean go_signal RT (M = 924, SD = 99; *F*(1,15) = 14.91, *p* = 0.002, *η* ^*2*^ = 0.50). In addition, we observed that SSRT values were similar to those observed in previous studies using a SST (neutral: M = 177, SD = 62; poker: M = 163, SD = 43; refs 44 and 45). Also we observed that the mean percentage of stimuli categorization accuracy was high (all means > 96%), and the mean percentage of miss acceptable (yellow: M = 0.09, SD = 0.06; orange: M = 0.10, SD = 0.08; red: M = 0.13, SD = 0.10). Further details on these data analyses are included in Supplementary Materials.

### In scanner behavior

#### Proactive inhibition

**Figure 1:**
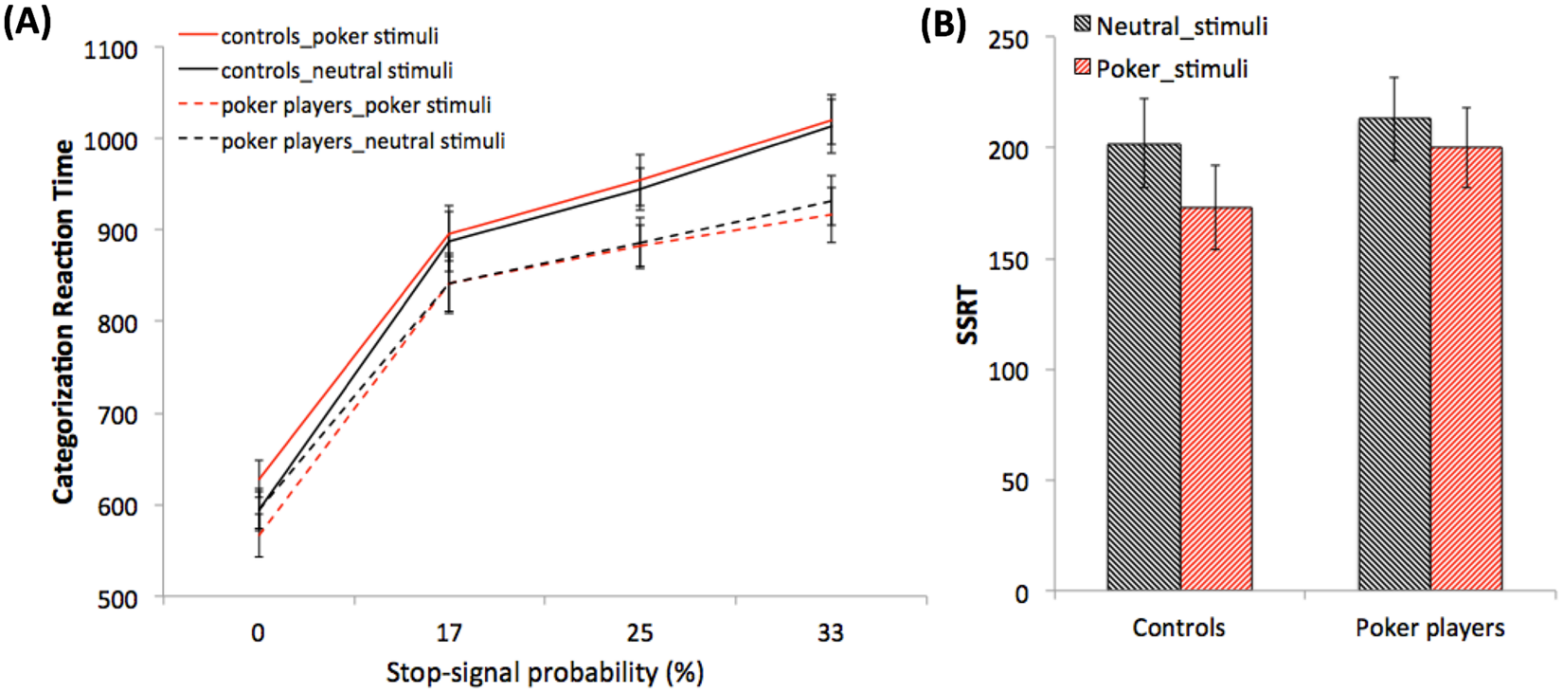
(**A**) Proactive inhibition: effect of stop-signal probability contexts (0% = green, 17% = yellow, 25% = orange, 33% = red) on go_signal response time for poker (in red) and neutral (in black) stimuli in the control and the poker player groups; (**B**) Reactive inhibition: stop-signal reaction time (SSRT) for poker (in red) and neutral (in black) stimuli in the control and the poker player groups. All errors bars indicate 95% confidence intervals.

Analyses revealed that categorization RT (in milliseconds) increased as a function of the level of stop-signal probability, *F*(3,26) = 161.38, *p* < 0.001, *η* ^*2*^ = 0.86 (see Fig. 1A; see also Supplementary Materials: Table S3 includes descriptive statistics on Go-signal RT, *p*(miss) and stimuli categorization accuracy; Table S4 includes mean RT on go-trials and mean SSD on stop-trials for each consecutive block of trials, separately for each stop-signal probability context, and averaged across the whole sample of participants, N = 28). Pairwise comparisons showed that there were significant RT increases between each context of stop-signal probability (red > orange > yellow > green; all *p* < 0.001). We observed a main effect of group, *F*(1,26) = 4.30, *p* = 0.048, *η* ^*2*^ = 0.14, indicating that poker players were faster in categorizing neutral and poker cues across all levels of stop-signal probability (green, yellow, orange, red). We observed no main effect of stimulus type and no significant interaction (all *p* > 0.09).

### Reactive inhibition

First, we observed that the overall sample mean of *p*[respond|signal]) was close to 0.50 for both the neutral (M = 0.52, SD = 0.07) and the poker (M = 0.53, SD = 0.06) stimuli, which confirm the effectiveness of the tracking procedure for both types of stimuli. We also observed that mean failed stop-signal RT was faster than mean go_signal RT, *F*(1,26) = 17.93, *p* < 0.0001, *η* ^*2*^ = 0.43, which is a criterion for the independence between the finish times of the *go* and the *stop* responses (see Supplementary Materials, which also include data analyses on stimuli categorization accuracy, *p*(miss), and *p*[respond|signal]). Repeated-measures ANOVA revealed that gamblers (neutral pictures: M = 213, SD = 63; poker pictures: *M* = 200, *SD* = 70) did not differ form controls (neutral pictures: *M* = 202, *SD* = 68; poker pictures: *M* = 172, *SD* = 66; see also Fig. 1B) on SSRT scores, *F*(1,26) = 0.69, *p* = 0.41, *η* ^*2*^ = 0.03. There was also no main effect of stimulus type or group, and no significant interaction effect (all *p* > 0.23).

### Brain activations during proactive inhibition, reactive inhibition, and cue reactivity

#### Proactive inhibition

**Figure 2:**
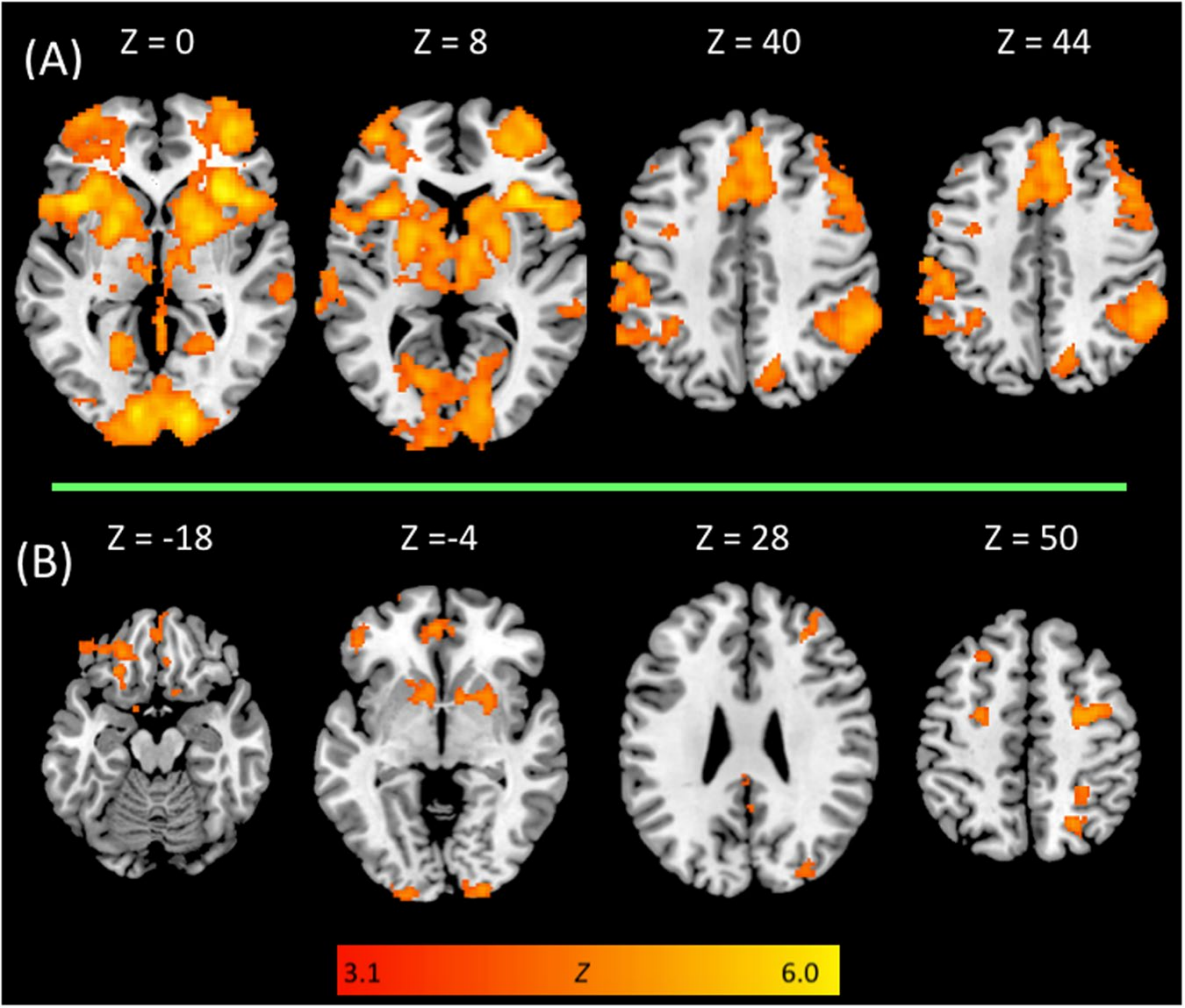
Whole-brain activation within the whole sample of control and poker player (*N* = 28) during (**A)** proactive motor response inhibition and (**B)** reactive motor response inhibition. All contrast maps were thresholded using cluster detection statistics, with a height threshold of z > 3.1 and a cluster probability of *p* < 0.05, family-wise error corrected for multiple comparisons.

Figure 2A and Tables S5 (in Supplementary Materials) show brain regions in which activation increased as a function of stop-signal probability on go-signal trials when categorizing poker and neutral pictures. This includes the whole sample of participants (*N* = 28). These contrasts reveal activations in a widespread network featuring frontal, paracingulate, insular, striatal, temporal, parietal, and occipital regions. However, no significant between-groups difference was observed on whole brain activation for the effect of stop-signal probability.

### Reactive inhibition

**Figure 3:**
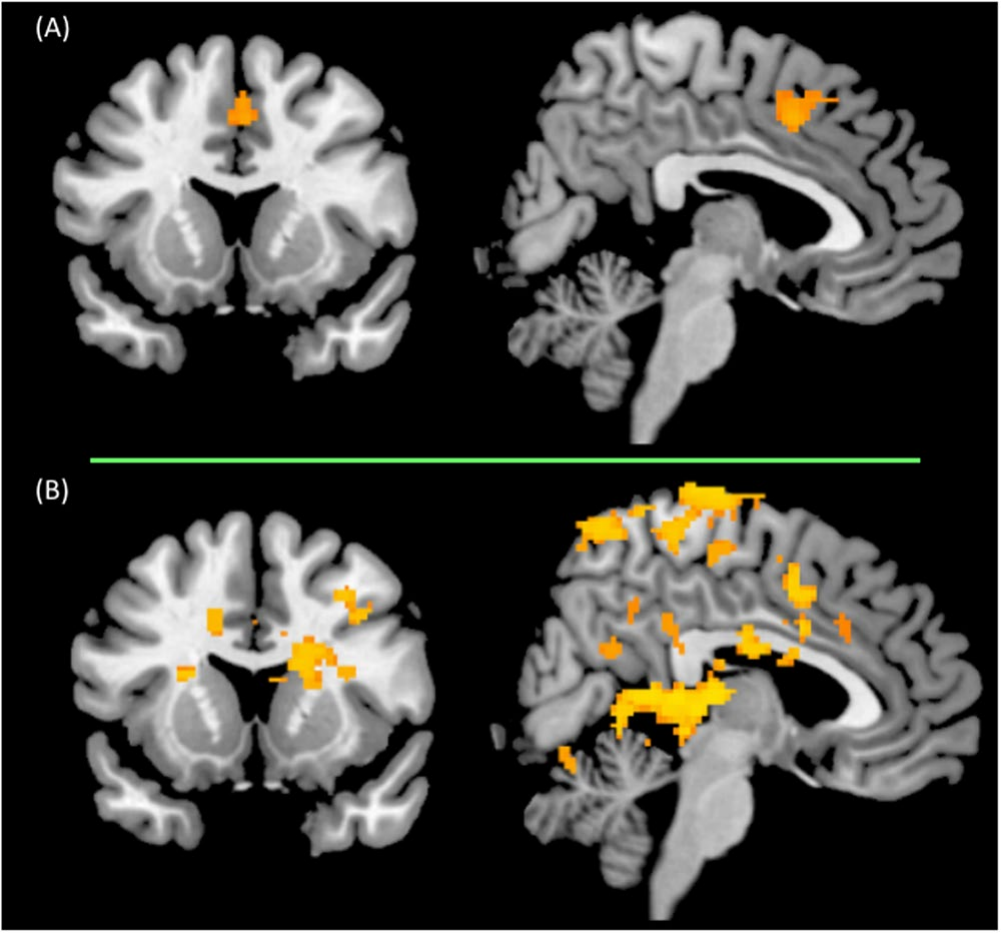
(**A**) Higher dorsal anterior cingulate activation (MNI coordinates: *x* = 4, *y* = 12, *z* = 44) during reactive inhibition (i.e., successful minus unsuccessful stop) in poker players, as compared to controls. **(B)** Higher brain activation in poker players, as compared to controls, for the successful stop minus baseline contrast. These contrast maps were thresholded using TFCE with *p* < 0.05, corrected for multiple comparisons across the whole brain.

Figure 2B and Tables S6 (in Supplementary Materials) show brain regions in which activation increased for the “successful stop minus unsuccessful stop” contrast, for the whole sample of participants (*N* = 28). Together, poker players and control subjects activated a network encompassing frontal, paracingulate, striatal, parietal and occipital regions. Between-groups analyses revealed that, when inhibiting successfully their motor response toward poker cues, poker players showed more activation than controls in the dorsal anterior cingulate cortex (Brodmann area = 32, cluster voxel size = 279, MNI coordinates: *x* = 4, *y* = 12, *z* = 44; see also Fig. 3A). No other between-group differences were observed.

In order to further emphasize the directionality of the between-group difference in dACC activity, two additional contrasts were created: successful stop minus baseline and unsuccessful stop minus baseline. Non-parametric permutation analyses (with 10,000 random permutations of the data, using TFCE thresholding with *p* < 0.05 corrected for multiple comparisons across the whole brain), were ran on these two contrasts. For the successful stop contrast, we observed that poker players exhibited higher brain activation than controls, including cluster of activation encompassing the dACC (see also Fig. 3B, and Table S7 in Supplementary Materials). No significant between-group difference was observed for the unsuccessful stop contrast.

Additional analyses were also undertaken in order to examine the relationship between dACC activity and stop-signal task performance. Specifically, we first extracted parameter estimates, for each participant, for the successful stop minus unsuccessful stop. The parameter estimates values were extracted from the dACC cluster obtained from non-parametric permutation analyses (TFCE thresholding with *p* < 0.05 corrected for multiple comparisons across the whole brain). We then ran Spearman Rho correlation analyses (*N* = 28; adjusted for multiple comparisons with Bonferroni correction) between parameter estimates and behavioral index of proactive and reactive inhibition. For proactive inhibition, we used the stop-signal probability slope^38^, defined as the change in go-signal response time per stop-signal probability unit increase. For reactive inhibition, we used (i) SSRT values, and (ii) the probability of responding on stop-signal, *p*(responding), averaged across all stop-signal probability contexts (i.e., yellow, orange, red). These analyses revealed a significant negative correlation between dACC parameter estimates, and the stop-signal probability slope, *rho*(28) = −0.47, *p* = 0.03. Thus, the lower the proactive adjustment is (i.e., faster stimuli categorization in stop-signal related contexts), the higher the dACC activity during the subsequent successful reactive inhibition. No significant correlation was observed between dACC parameter estimates with either SSRT, *rho*(28) = 0.11, *p* = 0.62, or *p*(responding), *rho*(28) = 0.36, *p* = 0.14. Noteworthy, there was also no correlation between SSRT and *p*(responding), *rho*(28) = 0.30, *p* = 0.15, which might be due to the fact that the SSRT (especially when estimated with the “integration method”; ref. 46) controls for variations in go-response reaction time, as well as in the proportion of incorrect inhibition.

#### Cue reactivity

During proactive inhibition, participants (*N* = 28) exhibited higher activation within the pre- and post-central gyrus. During successful reactive inhibition poker players and controls exhibited higher activation within the occipital pole. During unsuccessful reactive inhibition, participants (*N* = 28) exhibited higher activation within the pre- and post-central gyrus and the occipital pole. These activations are detailed in Tables S8 in Supplementary Materials. No significant between-groups activation was observed for all cue-reactivity contrasts.

## Discussion

In this study, we used fMRI to examine proactive (the restrain of actions in preparation for stopping) and reactive (a “late correction” process that results in the stopping of the ongoing action) motor response inhibition in a sample of highly frequent poker players and matched non-gambler controls. We used a modified version of the stop-signal paradigm that requested participants to inhibit categorizing poker and neutral pictures, and in which the probability that a stop-signal occurs was manipulated according to the color of the computer back screen.

At a behavioral level, we observed that poker players and non-gamblers exhibited comparable behavior of proactive (i.e., RT increase in function of the probability of stop-signal) and reactive (i.e., SSRT) motor response inhibition. However, poker players were faster than controls in categorizing poker and neutral cues during “go” trials (RT pooled across the green, yellow, orange and red contexts). This result is congruent with previous research, which showed that frequent gamblers (ranging from non-problem to high problem gambling) are faster in detecting gambling-related cues during rapid serial visual presentation tasks^47^. They also exhibit attentional bias toward gambling pictures at both early (attentional encoding and engagement; ref. 48) and late (maintenance and disengagement of attention; refs 48–54) levels of attentional processes. Interestingly, the faster pattern of stimulus categorization also encompassed neutral cues. One explanation for this finding is that, due to their expertise in discriminating poker cues on a daily-life basis, it might have been “easier” for high-frequent poker players to flag neutral pictures as “non-poker” cues (rather than neutral cues per se) during the go-trials of the stop-signal task. Another complementary explanation is that perceiving poker cues might have enhanced poker player involvement in the task, which might have impacted the speed accuracy on go-signal trials, e.g. ref. 55. In this context, because stimuli categorization is relative rather than absolute during the stop-signal task (i.e., right key pressing opposed to left key pressing), future studies should use a stop-signal task, with blocks of trials that include only neutral (e.g., discriminating between a table with or without chairs) or poker pictures (e.g., with money bills vs. without money bills). This procedure would allow disentanglement of (proactive and reactive) motor response inhibition of neutral and salient-motivational cues.

At a brain imaging level, within-group analyses revealed that contrasts for proactive and reactive motor response inhibition activated a widespread network that is very similar to that reported by previous brain imaging studies^29–40^. This includes frontal, insular, striatal, temporal, parietal, and occipital regions. In addition, both proactive and reactive inhibition activated the superior, middle, inferior, orbital frontal gyri, cingulate gyrus, superior parietal lobule, putamen and caudate. This neural pathway was activated in both the right and left hemispheres. These findings further confirm those from previous neuroimaging studies on proactive and reactive inhibition in showing that these two modes of response control share a common brain network^36^. This common neural network might be involved in “braking” motor output when it is weakly activated (proactive inhibition), and in “stopping” motor output completely when strongly activated (reactive inhibition)^26, 36^. Importantly, between-group analyses showed that poker players exhibited higher brain activation than controls during reactive inhibition (i.e., the “successful minus unsuccessful stop” contrast). This pattern of activation was revealed within the dACC. This region plays a key role in cognitive control by monitoring the occurrence of response conflict^56–58^, such as when simultaneous incompatible response tendencies are triggered during reactive inhibition on the stop-signal task (i.e., a “go” process triggered by the go stimulus that races against a stop process triggered by the “stop signal”; refs 46, 59, 60). This differential pattern of activation might be explained by the poker players’ faster stimuli categorization speed (as compared to non-gambler control participants), which was observed at the behavioral level of analyses. Specifically, due to quicker go responses, poker players might have needed more cognitive resources (as evidenced with higher dACC activation) than controls when required to cancel their motor response on stop trials, i.e., during successful reactive motor response inhibition. This assumption is in line with correlation analyses, which showed that dACC parameter estimates from the reactive inhibition contrast (i.e., successful minus unsuccessful stop) was significantly associated with behavioral measures of proactive control (i.e., the stop-signal probability slope, which is estimated with go-trial RTs), but not with reactive control (either *p*[responding] or SSRT). In other words, the lower the proactive adjustment is (i.e., faster stimuli categorization in stop-signal related contexts), the higher the cognitive resources needed during subsequent reactive inhibition.

One major implication of this finding is that, because reactive motor response inhibition is a high-demanding process^26, 29, 30, 61, 62^, and that poker gamblers might rely on more effortful-reactive inhibitory control, they should be less able to undertake repeated and extended inhibitory effort during poker cues exposure. This “backfiring” pattern could relate to a mechanism of ego depletion, which has been reported to occur when an extended inhibitory effort in one domain causes subsequent inhibitory impairment in a second domain^63^. For instance, Muraven and colleagues^64^ found that an episode of affective inhibition resulted in a subsequent reduction in the ability to exercise motor control, and that prolonged cognitive inhibition resulted in a deficit in inhibiting emotional stimuli.

Contrary to our hypotheses, we observed no significant impact on the type of stimuli during either proactive or reactive motor response inhibition. Specifically, at a behavioral level, poker cues did not impact poker players proactive RT slowing and SSRT. At a neural level, cue reactivity contrast revealed that poker stimuli activated mainly the primary motor cortex, i.e., the brain network involved in overt motor action during go trials (i.e., the pressing of the response key). One explanation for this negative finding is that the action of poker gambling was not perceived as “available” or “expected” by poker gamblers throughout the experiment. In other words, in the current study, the stop-signal task was not followed by the action of poker playing. Hence, the viewing of poker pictures might not have triggered strong motivational-approach in poker players because the stimulus “complex” was not complete. Consistent with this account, perceived availability has been shown to increase craving in smokers^65, 66^ and social drinkers^67^, and could lead the individual to respond more forcefully to salient stimuli^19, 68^. At a brain-imaging level, it has been shown that stimulus availability and expectancy increases neural cue reactivity (for a review, see ref. 69). For instance, Blechert and colleagues^70^ showed that viewing available, compared to unavailable, food cues elicited stronger neural activation in structures implicated in reward and appetitive motivation (amygdala, caudate nucleus), as well as cognitive control (anterior cingulate cortex, orbitofrontal cortex, medial prefrontal cortex). Thus, one option to expose the effect of stimulus type on (proactive and reactive) motor response inhibition would be to manipulate perceived availability attached to the action of poker (e.g., to make participant believing that he will have the opportunity to participate in a poker gambling session directly after having performed the stop-signal task). Alternatively, future studies should focus on the impact of addiction-related cues in quitting-motivated addicts. Indeed, it has been shown that response inhibition toward food cues is enhanced in overweight adults who are attempting dietary restrain^22, 71, 72^. Similar findings have been reported in treatment-seeking addicted individuals, who exhibited intact^73^, or enhanced^15^ motor response inhibition towards drug or gambling addition related-cues, when compared to non-addicted controls. This can be accounted for, if one assumed that abstaining or restraining gamblers, in contrast to the current frequent gamblers, develop an active avoidance strategy toward cues to support their abstinence/moderation goals^18, 69^. Another important point is that our modified version of the stop-signal task involved explicit manipulation of stop-signal probability (i.e., the present task is cognitively more complex than the standard stop-signal task). Hence, despite the validation indices that were obtained for reactive inhibition (i.e., *p*[respond|signal]) close to 0.50; mean failed stop-signal RT faster than mean go_signal RT; mean SSRT values similar than in studies using a standard stop-signal task) and proactive inhibition (linear increases in RT slowing in function of stop-signal probability), the complexity of the present design might have decreased the influence of motivational cues on response inhibition. In addition, the use of a high value for the initial SSD (i.e., 800 ms) might have “forced” participants to wait for stop-signal (e.g., the picture categorization reaction time in the 33% condition is almost twice longer than in the 0% condition). This might have influenced the optimal assessment of motor response inhibition in the current stop-signal task, which is a paradigm that requires participants to respond fast, and not to wait for stop-signals. Hence, future studies should examine proactive and reactive inhibition using shortened initial SSD value and/or assess whether these two components of inhibition are significantly modulated (at both behavioral and neural levels) when different initial SSD values are used.

Finally, because the sample sizes of our groups were modest, non-significant behavioral and neuroimaging results must be interpreted with caution. Nevertheless, our final sample is comparable to that in van Holst *et al*. study^15^, i.e., another neuroimaging study that examined the impact of gambling cues on motor response inhibition in gamblers. Furthermore, our sample was selected using stringent selection criteria, resulting in a homogeneous cohort of gamblers in terms of gambling game preferences (i.e., poker).

In summary, the present findings indicate that, compared to non-gambler controls, highly frequent poker players show more activation of the dorsal anterior cingulate cortex during reactive response inhibition of neutral and poker pictures. This pattern might be due to poker players’ faster rates of stimulus discrimination exhibited across varying levels of proactive motor response inhibition. The present results confirm that investigating both reactive and proactive inhibition might offer a discriminative and fine-grained analysis of inhibitory control, and that fMRI is a good approach for studying the dynamics underlining these two processes. To a broader extent, these findings suggest that humans need to trigger additional cognitive resources, when required to stop their motor response, while being embedded in an environment featuring salient-motivational stimuli. Examining the dynamics between proactive and reactive control could, therefore, advance our knowledge of humans’ ability to control hedonic habits, and further inform the development of novel intervention strategies aimed at strengthening self-control.

## Methods

### Participants

Seventeen frequent poker gamblers (11 males and 6 females, age 28.91 years, education level 14.02 years) and 16 non-gambler controls participated in this study (10 females and 6 males, age 26.87 years, education level 16.58 years). All participants gave written informed consent to the experimental procedure, which was approved by the University of Southern California Institutional Review Board and this study was performed strictly in accordance with the approved guidelines.

Poker players were recruited on the Internet through advertisements displayed on online forums for poker players based in Los Angeles. The ads asked for participants who “played poker frequently in casino” to participate in a one-day study to explore factors associated with attentional processing in poker gambling. Controls were recruited by word of mouth from the community. For both gamblers and controls, a screening interview was conducted (online or through the phone) in order to examine gambling frequency, problem gambling severity, history of therapeutic intervention focused on gambling behavior, substance use (alcohol drinks per day), and medical history (included in the MRI screening form). Problem Gambling severity was assessed with the South Oaks Gambling Screen (SOGS). Current sample of gamblers ranged from non-problem to high-problem gambling. Poker players’ SOGS scores and information on their frequency of poker playing (per week) is depicted in Supplementary Materials, Table S1. None of the gambler participants reported a history of therapeutic treatment focused on gambling behavior. Controls were excluded if they gambled frequently (i.e., more than 2 times per month) or if they scored 2 or higher on the SOGS. Participants were also excluded if they reported excessive substance use (e.g., an average of three drinks or more per day over the last year). All subjects were judged to be physically healthy on the basis of their answers on the MRI screening form. Participants were advised to avoid alcoholic drinks in the 24 h prior to testing. All subjects were right handed and had normal or corrected-to-normal vision.

### The Stop-Signal Task

Participants performed a modified stop-signal task (SST; see Fig. 1), a paradigm adapted from previous stop-signal tasks^37, 38, 74^. In this task, participants had to discriminate, as fast as they can, between neutral and poker-related pictures. Subjects were asked to stop their response when they heard a tone (stop-signal). During the experiment, stop-signal delay (SSD; the interval between trial onset and the presentation of the stop-signal) was continuously adjusted (initial value = 800 ms), separately for neutral and gambling cues (across stop-signal probability contexts; i.e., yellow, orange, red), according to a tracking procedure to obtain a probability of stopping of 0.50^46, 59, 60^ if stopping was successful, then stopping was made more difficult on the next stop-trial by increasing SSD by 25 ms. The process was reversed when stopping failed.

The probability that a stop-signal occurs was manipulated across trials and was indicated by the color of the computer back screen: 0% (green), 17% (yellow), 25% (orange), and 33% (red). Trials were divided into blocks of 9, 18 or 27 trials in a same context. Block length was randomized with the restriction that there were no context repetitions (e.g., 2 × green context trials, or 2 × 9 green context trials) and that blocks of 9, 18 and 27 trials occurred with equal probability.

**Figure 4:**
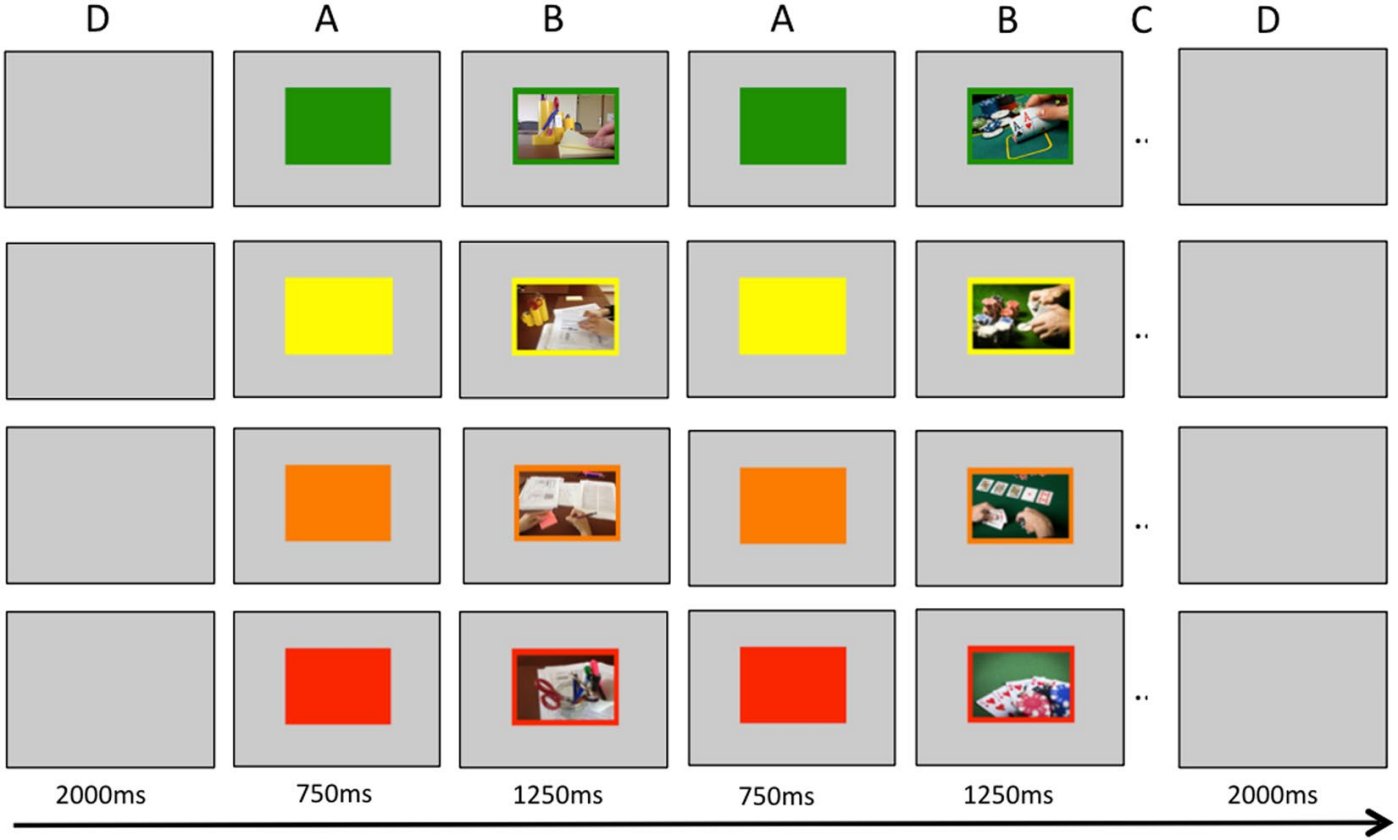
An example of a succession between a neutral and a poker pictures in the green, yellow, orange and red contexts of the stop-signal task. (**A**) Each trial started with the presentation of the context cue for 750 ms. (**B**) A picture then appeared during 1250 ms, regardless of participants’ categorization reaction time. (**C**) Trials were divided into runs of 9, 18 or 27 trials in a same context. (**D**) A context change was indicated by a 2000 ms grey screen.

Each trial started with the presentation of the context cue for 750ms (Fig. 4A). Each picture then appeared during 1250 ms (Fig. 4B), regardless of participants’ categorization reaction time. Each context change was separated by a 2000ms grey screen (Fig. 4D). In total, 350 go-signal trials (0%, *n_neutral* = 64, *n_poker* = 64; 17%, *n_neutral* = 45, *n_poker* = 45; 25%, *n_neutral* = 40, *n_poker* = 40; 33%, *n_neutral* = 36, *n_poker* = 36) and 82 stop-signal trials (17%, *n_neutral* = 9, *n_poker* = 9; 25%, *n_neutral* = 14, *n_poker* = 14; 33%, *n_neutral* = 18, *n_poker* = 18) were presented in a single run in pseudorandom order. Poker pictures were taken from casino scenes and were matched on visual complexity with the neutral pictures (for an illustration, see Fig. 4). We used 18 poker and 18 matched neutral pictures. Each picture was repeated for 12 times, which was randomly distributed across the green, yellow, orange and red contexts.

### MRI Procedure and Data Acquisition

Participants were instructed to categorize pictures as soon as possible, unless they heard a “beep” sound while the picture appears on the screen. They were informed that the Go task and Stop task were equally important and that it would not always be possible to suppress a response when a stop-signal occurred. We informed participants that stop-signals would never appear on trials with a green cue and that stop-signals could occur on trials with non-green cues. Participants were told that stop-signals were least likely in the context of a yellow cue and most likely in the context of a red cue, with the orange cues coding intermediate stop-signal probabilities. This instruction procedure was based on previous work by Zandbelt and colleagues^37, 38^.

Before entering the MRI scanner, participants were first trained on the SST in order to familiarize them with the task. One experimenter remained alongside the participants during the training in order to ensure that the task was properly understood. The training consisted of 9 trials for each of the four background color contexts (total of 36 trials). During the MRI scanning session, participants lay supine on the scanner bed, and viewed visual stimuli back-projected onto a screen through a mirror attached onto the head coil. Foam pads were used to minimize head motion. Stimulus presentation and timing of all stimuli and response events were achieved using Matlab 7.14 (Mathworks Inc., Natick, MA, USA) and Psychtoolbox 3.0.12 (www.psychtoolbox.org) on an IBM compatible PC. Participants’ responses were collected online using an MRI-compatible button box.

fMRI imaging was conducted in a 3 T Siemens MAGNETOM Prisma scanner in the Dana and David Dornsife Cognitive Neuroscience Imaging Center at the University of Southern California. Functional scanning used a z-shim gradient echo EPI sequence with PACE (prospective acquisition correction). This specific sequence is dedicated to reduce signal loss in the prefrontal and orbitofrontal areas. The PACE option can help reduce the impact of head motion during data acquisition. The parameters are: TR = 2000 ms; TE = 25 ms; flip angle = 90°; 64 × 64 matrix size with resolution 3 × 3 mm^2^. Thirty-one 3.5 mm axial slices were used to cover the whole cerebral cortex and most of the cerebellum with no gap. The slices were tilted about 30 degree clockwise along the AC-PC plane to obtain better signals in the orbitofrontal cortex. The anatomical T1-weighted structural scan was done using an MPRAGE sequence (TI = 800 ms; TR = 2530 ms; TE = 3.1 ms; flip angle 10°; 208 sagittal slices; 256 × 256 matrix size with spatial resolution as 1 × 1 × 1 mm^3^).

### Image Preprocessing

Image preprocessing was carried out using FEAT (FMRI Expert Analysis Tool) version 6.00, part of the FSL package (FMRIB software library, version 5.0.9, www.fmrib.ox.ac.uk/fsl). The first three volumes, before performance of the task, were automatically discarded by the scanner to allow for T1 equilibrium. The remaining images were then realigned to compensate for small residual head movements that were not captured by the PACE sequence^75^. Translational movement parameters never exceeded 1 voxel in any direction for any subject or session. Data were spatially smoothed using a 5-mm full-width-half-maximum (FWHM) Gaussian kernel. The data were filtered in the temporal domain using a non-linear high pass filter with a 100-s cut-off. A three-step registration procedure was used whereby EPI images were first registered to the matched-bandwidth high-resolution scan, then to the MPRAGE structural image, and finally into standard (MNI) space, using affine transformations^75^. Registration from MPRAGE structural image to standard space was further refined using FNIRT nonlinear registration^76, 77^. Statistical analyses were performed in the native image space, with the statistical maps normalized to the standard space prior to higher-level analysis.

### Behavioral Data Analysis

Behavioral data were analyzed using custom written software in Matlab 7.14 (Mathworks Inc., Natick, MA, USA) and SPSS 24 (SPSS, Inc., Chicago, IL, USA). Response times (for Go trials) and accuracy were calculated for each stop-signal probability level separately. In keeping with previous studies^36, 38–40, 46^, proactive inhibition was measured as the effect of stop-signal probability on go-signal response time. Typically, subjects tend to slow down responding as the probability that they may have to stop increases. Reactive inhibition was estimated in terms of stop-signal reaction time (SSRT), which is a measure of the latency of the inhibition process. The SSRT was estimated through the integration method^46^ and pooled across stop-signal probability levels^38, 39^. The integration method involves to subtract the mean SSD from *n*th RT (with *n* equal to the number of RTs in the RT distribution; missed responses were included and set to 1250 ms, that is, the response deadline) multiplied by the overall *p* [respond|signal]). The SSRT was estimated separately for neutral and gambling pictures. Go trials with response times of more than 1.5 times the interquartile range away from the 25th and 75th percentiles of the response time distribution of each stop-signal probability level were defined as outliers.

Due to poor task performance, data from three gamblers and one control were excluded. These participants exhibited more than 70% of go response during stop-signal trials (i.e., due to the tracking procedure, percentage of incorrectly executed go responses should be close to 50%, regardless of the latencies of the go and stop processes). Another control participant was excluded due to a technical issue (no stop-signal « beep » sound during the second half of the task) yielding 14 gamblers and 14 controls for behavioral and fMRI analyses. Statistical analysis of proactive inhibition consisted of a repeated-measures analysis of variance (ANOVA) on mean go-signal response times in each stop-signal probability context (green, yellow, orange, red), stimulus type (neutral versus poker), and group (control vs. poker players) as factors. Statistical analysis of reactive inhibition involved repeated-measures ANOVA on SSRT, with stimulus type (neutral versus poker), and group (control vs. poker players) as factors.

### Brain Imaging Analyses

The brain imaging data were modeled at the first level using a general linear model within FSL’s FILM module using FEAT. First-level statistical analysis included the following conditions as regressors: successful stop-signal trials, unsuccessful stop-signal trials, go-signal trials stop-signal probability levels (green, yellow, orange, red), for both poker and neutral pictures (i.e., 6 × 2 = 12 conditions of interest in total). The event onsets were convolved with canonical hemodynamic response function (HRF, double-gamma) to generate regressors used in the GLM. Temporal derivatives and missed go responses for go-signal trials were included as covariates of no interest to improve statistical sensitivity. Null events were not explicitly modeled, and therefore constituted an implicit baseline.

For each participant, we computed the following contrast images: (i) To assess reactive inhibition: activation during successful stop-signal trials minus unsuccessful stop-signal trials; (ii) To assess proactive inhibition: a parametric contrast on go trials with the four stop-signal probability contexts (green, yellow, orange, red) coded as mean-centered probabilities; iii) to assess poker cue reactivity during proactive inhibition: parametric modulation by probability during poker cue go trials minus parametric modulation by probability during neutral cue (go trials); iv) to assess poker cue reactivity during reactive inhibition: successful stop during poker cue (stop-signal trials) minus successful stop during neutral cue (stop-signal trials), and unsuccessful stop during poker cue (stop-signal trials) minus unsuccessful stop during neutral cue (stop-signal trials). On each set of contrast images, we performed two group analyses. First, whole-brain activations were visualized for the whole sample (*N* = 28), using one-sample *t*-tests. Maps resulting from these group analyses were tested with random-effect model for group analysis using FLAME 1^78–80^ and thresholded using cluster detection statistics, with a height threshold of z > 3.1 and a cluster probability of *p* < 0.05, family-wise error corrected for multiple comparisons. Second, between-groups differences in whole-brain activation were tested using non-parametric permutation methods for inference on statistic maps (Randomise v2.1 in FSL; see http://fsl.fmrib.ox.ac.uk/fsl/fsl-4.1.9/randomise/index.html) with 10,000 random permutations of the data. Maps resulting from between-groups analyses were thresholded using Threshold-free cluster enhancement (TFCE) with *p* < 0.05 corrected for multiple comparisons across the whole brain.

## Electronic supplementary material

Supplementary Materials
