# Supplementary material for: Neural correlates of proactive and reactive motor response inhibition of gambling stimuli in frequent gamblers
Source: Sci Rep. 2017 Aug 7;7:7394. doi: 10.1038/s41598-017-07786-5 (PMC5547049; doi:10.1038/s41598-017-07786-5)
Supplement: Supplementary file 1 — Supplementary Materials [file 41598_2017_7786_MOESM1_ESM.doc]

**Neural correlates of proactive and reactive motor response inhibition of gambling stimuli in frequent gamblers**

Brevers, D.^a^ ^b^, He, Q.^c^, Keller, B.^a^, Noël, X.^b^, Bechara, A^a^.

^a^Department of Psychology, and Brain and Creativity Institute, University of Southern California, Los Angeles, CA, USA

^b^Psychological Medicine laboratory, Faculty of Medicine, Brugmann-campus, Université Libre de Bruxelles, Brussels, Belgium

^c^Faculty of Psychology, Southwest University, 2 Tiansheng Rd, Chongqing, China

*Corresponding author: Brevers Damien, PhD, Brain and Creativity Institute, University of Southern California, 3620A McClintock Avenue, 90089-2921, Los Angeles, CA, USA

Email: brevers@usc.edu

***Supplementary Information***

**Notes**

***Selection of initial SSD value.*** 800ms was used as the SSD initial value based on repeated observations made during pilot testing of the task (i.e., before running the behavioral task validation included in the manuscript).

Specifically, on the one hand, we observed that the probability of response on stop-signal, *p*(responding), was not-optimal (e.g., < .40) when using shorter initial value such as 250 or 550ms (i.e., the first half of the stop-task was too easy). This might be explained by the visual complexity of the stimuli to be categorized (matched neutral or poker cues depicting a scene featuring multiple components). Accordingly, stimuli categorization RT in the green context (i.e., the response inhibition-free context) was around 600ms. Noteworthy, in one of our current experiment using similar SST but with less complex stimuli categorization (right vs. left arrows), an initial SSD value of 500ms was optimal for obtaining a mean *p*[respond|signal]) (pooled across the yellow, orange, and red contexts) that approximate .50. On the other hand, when using higher initial SSD value (e.g., 900), we observed abnormally high percentage of missed response on go trials (> .20 across all task). In sum, when using 800ms as initial SSD value, we obtained a mean *p*[respond|signal]) (pooled across the yellow, orange, and red contexts) approximating .50, and with an acceptable level of miss on go trials (< 10 % across all trials).

***SST design.*** Response inhibition during stop-signal performance is typically described as a race between a go process, triggered by a go stimulus, and a stop process, triggered by the stop signal. In this context, we used fixed ITI, so that the onset of each target stimulus (i.e., a neutral or a poker picture) was perceived as constant by the participant. This aspect was crucial in order not to interfere with the speed of the “go process” attached to stimuli categorization, which allowed obtaining accurate estimations of proactive and reactive inhibition. In other words, together with the occurrence of the stop-signal (which was continuously adjusted according to a tracking procedure and with a probability of apparition that differed under the green, yellow, orange and red contexts), a variable ITI would have added some more uncertainty related to the onset of the “go” response (i.e., categorizing neutral and poker cues) and would have biased our measures of response inhibition.

Moreover, in order to optimize the impact of each context of stop-signal probability (i.e., green, yellow, orange, red) on proactive inhibition, we divided trials into blocks of 9, 18 or 27 trials in a same context (participants were informed that each context change occurred when a grey screen appeared). Indeed, in a pilot version of the task, we observed that reaction time difference between the different contexts of stop-signal probability was lower when the background color varied from trial to trial. The proportion of misses was also increased. One explanation is that changing the background color on each trial of the stop-signal task required the participant to reinitiate context identification on every trial, which might have lowered proactive adjustment between each context of stop-signal probability in our stop-signal task.

**Participants**

Gamblers were recruited on the Internet through advertisements displayed on online forums for poker players based in Los Angeles. The ads asked for participants who ‘‘played poker frequently in casino’’ to participate in a one-day study to explore factors associated with attentional processing in poker gambling. Controls were recruited by word of mouth from the community. For both gamblers and controls, a screening interview was conducted (online or through the phone) in order to examine gambling frequency, problem gambling severity, history of therapeutic intervention focused on gambling behavior, substance use (alcohol drinks per day), and medical history (included in the MRI screening form). Problem Gambling severity was assessed with the South Oaks Gambling Screen. Current sample of gamblers ranged from non-problem to high-problem gambling. Gamblers’ SOGS scores and information on their frequency of poker playing (per week) is depicted in Supplementary Materials, Table S1. None of the gambler participant reported a history of therapeutic treatment focused on gambling behavior. Controls were excluded if they gambled frequently (i.e., more than 2 times per month) or if they scored 2 or higher on the SOGS. Participants were also excluded if they reported excessive substance use (e.g., an average of three drinks or more per day over the last year). All subjects were judged to be physically healthy on the basis of their answers on the MRI screening form. Participants were advised to avoid alcoholic drinks in the 24h prior to testing.

**Behavioral task validation**

The SST was first pretested in a sample of students. Those pretests aimed to test whether categorization RT is modulated by the level of stop-signal probability (green < yellow < orange < red). In addition, we expected that (i) mean *p*[respond|signal]) (pooled across the yellow, orange, and red contexts) will approximate .50, which would confirm the effectiveness of the tracking procedure; and (ii) mean failed stop-signal RT should be faster than mean go signal RT (both measures pooled across the yellow, orange, and red contexts), which would be used as a criteria of independence between the finish times of the *go* and the *stop* responses.

***Participants.*** Sixteen students (8 males, 8 females, mean age = 19.92) from the University of Southern California (USC), College of Letters, Art, and Sciences participated in these pretests. After having signed an IRB-approved informed consent form, participants received the task-instructions, performed a practice session and then the main SST.

***Stimuli categorization accuracy.*** Mixed-model ANOVA were used with level of stop-signal probability (green, yellow, orange, red) and stimulus type (neutral vs. poker) as within-subjects factors; and proportion of correct stimuli categorization as dependent measure (see Table S2 for descriptive statistics). These analyses revealed a main effect of level of stop-signal probability, *F*(3,15) = 4.53, *p* = .008, *η²* = .23, indicating that proportion of correct stimuli categorization was higher in the yellow and the orange contexts. There was no significant effect of stimulus type, *F*(1,15) = 0.13, *p* = .72, *η²* = .01, and no significant response type × level of stop-signal probability interaction, *F*(3,15) = 1.51, *p* = .22, *η²* = .09.

***Go RT according to the level of stop-signal probability*.** Mixed-model analysis of variance (ANOVA) were used with level of stop-signal probability (green, yellow, orange, red) and stimulus type (neutral vs. poker) as within-subjects factors; and go_signal RT as dependent measure (see Table S2 for descriptive statistics). These analyses revealed a main effect of stop-signal probability levels, *F*(3,15) = 106.55, *p* < .0001, *η²* = .88, indicating that categorization RT increased in function of the level of stop-signal probability (see Figure S1). Pairwise comparisons revealed that there was a significant RT difference between the yellow_go and red_go (*p* < .0001), yellow_go and orange_go (*p* = .008), and orange_go and red_go (*p* = .001) conditions. There was no main effect of stimulus type, *F*(1,15) = 0.413, *p* = .53, *η²* = .03, and no significant interaction effect between the type of stimuli and the level of stop-signal probability, *F*(3,15) = 1.11, *p* = .36, *η²* = .07.

***Proportion of missed responses according to the level of stop-signal probability*.** Mixed-model ANOVA were used with level of stop-signal probability > 0% (yellow, orange, red) and stimulus type (neutral vs. poker) as within-subjects factors; and proportion of missed responses as dependent measure (see Table S2 for descriptive statistics). These analyses revealed no main effect of stop-signal probability, *F*(2,15) = 2.86, *p* = .007, *η²* = .16, and stimulus type, *F*(1,15) = 0.02, *p* = .94, *η²* = .00. There was also no interaction between the type of stimuli and the level of stop-signal probability, *F*(2,15) = 0.18, *p* = .83, *η²* = .012.

***Difference between mean failed stop-signal RT and mean go signal RT.*** Mixed-model ANOVA were used with stimulus type (neutral vs. poker) and response type (failed stop-signal RT vs. go_signal RT pooled across all stop-signal probability levels) as within-subjects factors; and RT as dependent measure. These analyses revealed a main effect of response type, *F*(1,15) = 14.91, *p* = .002, *η²* = .50, indicating that RT (in ms) was lower for failed stop-signal (M = 857, SD = 87) than for go_signal RT (M = 924, SD = 99). There was no significant effect of stimulus type, *F*(1,15) = 1.46, *p* = .24, *η²* = .08, and no significant response type × stimulus type interaction, *F*(1,15) = 0.11, *p* = .74, *η²* = .01.

***Stop Signal reaction Time (SSRT).*** Mixed-model ANOVA were used with stimulus type (neutral vs. poker) as within-subjects factors; and SSRT as dependent measure. These analyses revealed no difference between neutral (M = 177, SD = 62) and poker (M = 163, SD = 43) stimuli main effect of stimulus type, *F*(1,15) = 1.19, *p* = .29, *η²* = .07.

***Probability of responding on stop-signal trials according the level of stop-signal probability.*** Mixed-model ANOVA were used with level of stop-signal probability > 0% (yellow, orange, red) and stimulus type (neutral vs. poker) as within-subjects factors; and probability of responding on stop-signal trials as dependent measure (see Table S2 for descriptive statistics). These analyses revealed a main effect of level of stop-signal probability, *F*(2,15) = 22.21, *p* < .001, *η²* = .60, indicating that probability of responding on stop-signal trials decrease in function of stop-signal probability. There was no significant effect of stimulus type, *F*(1,15) = 1.34, *p* = .27, *η²* = .08, and no significant response type × level of stop-signal probability interaction, *F*(2,15) = 0.44, *p* = .64, *η²* = .03.

***Summary of pretests analyses***. Taken together, pretests analyses confirmed that: categorization RT (in ms) increased in function of the level of stop-signal probability (see also Figure S1); that mean *p*[respond|signal]) was close to .50 for both the neutral and the poker stimuli; and that the mean failed stop-signal RT was lower than the. In addition, we observed that SSRT values was similar than those observed in previous studies using a SST (neutral: M = 177; poker: M = 163), that the mean percentage of stimuli categorization accuracy was high (all mean > 96%), and the mean percentage of miss acceptable (yellow: M = .09, SD = .06; orange: M = .10, SD = .08; red: M = .13, SD = .10).

**In scanner behavior**

***Proportion of missed responses according to the level of stop-signal probability*.** Mixed-model ANOVA were used with level of stop-signal probability > 0% (yellow, orange, red) and stimulus type (neutral vs. poker) as within-subjects factors; groups (controls vs. gamblers) as between-subjects factor; and proportion of missed responses as dependent measure (see Table S3 for descriptive statistics). These analyses revealed a main effect of stop-signal probability, *F*(2,26) = 20.78, *p* < .0001, *η²* = .46, indicating that the proportion of miss increased with the level of stop-signal probability. There was no main effect of group, stimulus type or any significant interaction (all *p* > .14).

***Difference between mean failed stop-signal RT and mean go signal RT.*** Mixed-model ANOVA were used with stimulus type (neutral vs. poker) and response type (failed stop-signal RT vs. go signal RT pooled across all stop-signal probability levels) as within-subjects factors; groups (controls vs. gamblers) as between-subjects factor; and RT as dependent measure. These analyses revealed a main effect of response type, *F*(1,26) = 61.71, *p* < .0001, *η²* = .71, indicating that RT was lower for failed stop-signal (M = 807, SD = 107) than for go_signal RT (M = 907, SD = 130). There was no main effect of group, stimulus type or any significant interaction (all *p* > .12).

***Stimuli categorization accuracy.*** Mixed-model ANOVA were used with level of stop-signal probability (green, yellow, orange, red) and stimulus type (neutral vs. poker) as within-subjects factors; groups (controls vs. gamblers) as between-subjects factor; and proportion of correct stimuli categorization as dependent measure (see Table S3 for descriptive statistics). These analyses revealed a main effect of level of stop-signal probability, *F*(3,26) = 4.53, *p* = .008, *η²* = .23, indicating that proportion of correct stimuli categorization was higher in the yellow and the orange contexts. No other significant result was observed (all *p* > .13).

***Probability of responding on stop-signal trials according the level of stop-signal probability.*** Mixed-model ANOVA were used with level of stop-signal probability > 0% (yellow, orange, red) and stimulus type (neutral vs. poker) as within-subjects factors; groups (controls vs. gamblers) as between-subjects factor; and probability of responding on stop-signal trials as dependent measure (see Table S3 for descriptive statistics). These analyses revealed a main effect of level of stop-signal probability, *F*(2,26) = 38.50, *p* < .0001, *η²* = .61, indicating that probability of responding on stop-signal trials decrease in function of stop-signal probability. There was no main effect of group or stimulus type (all *p* > .11).

**
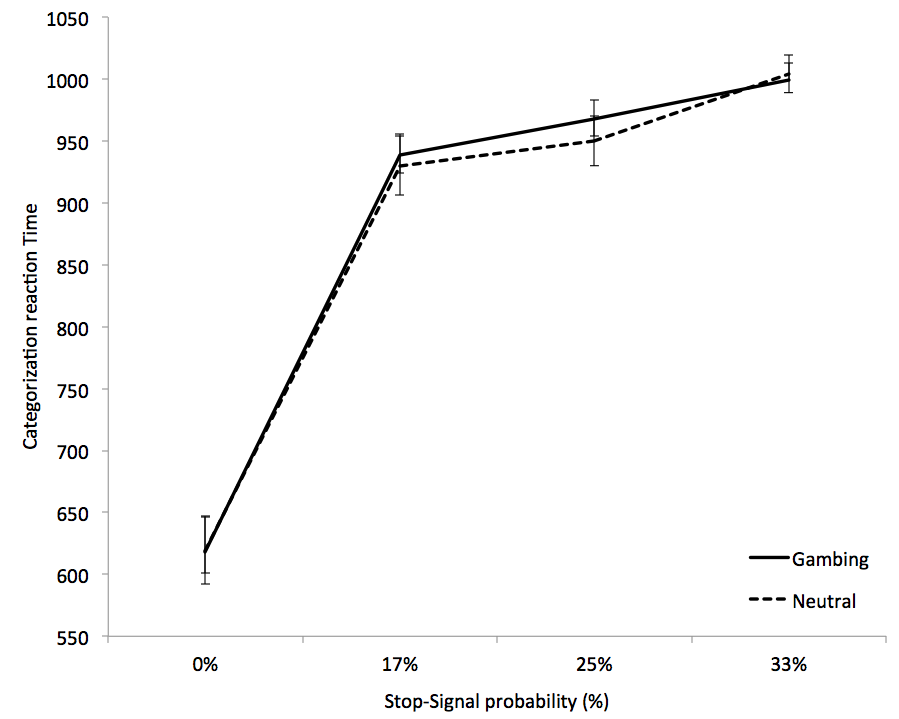
**

**Figure S1***.* Effect of stop-signal probability on go_signal response time for gambling and neutral pictures. Errors bars indicate 95% confidence intervals.

**Table S1. SOGS score, poker playing frequency, in the gambler group (*n* = 17).**

| SOGS score (average) | *M* = 4.67, *SD* = 3.23 |
| --- | --- |
| No problem with gambling (SOGS score = 0) | *n* = 2 |
| Some problem with gambling (SOGS score ranging from 1 to 4) | *n = 7* |
| Probable gambling disorder (SOGS score ≥ 5) | *n* = 8 |
| Poker playing frequency (day per week) | *M* = 4.79, *SD* = 2.25 |

SOGS, South Oaks Gambling Screen; *M*, mean; *SD*, Standard Deviation; *n*, number of subject.

| Level of *p*(stop-signal) | Stimulus type | *p*(correct) | |  | Go RT | |  | *p*(miss) | |  | *p*(respond|signal) | |
| --- | --- | --- | --- | --- | --- | --- | --- | --- | --- | --- | --- | --- |
|  |  | M | sd |  | M | sd |  | M | sd |  | M | sd |
| 0 | neutral | 0.96 | 0.03 |  | 618 | 109 |  | 0.01 | 0.02 |  | / | / |
| 0 | gambling | 0.97 | 0.02 |  | 619 | 113 |  | 0.02 | 0.04 |  | / | / |
| 17 | neutral | 0.98 | 0.01 |  | 931 | 84 |  | 0.09 | 0.06 |  | 0.57 | 0.11 |
| 17 | gambling | 0.98 | 0.02 |  | 938 | 85 |  | 0.10 | 0.07 |  | 0.60 | 0.15 |
| 25 | neutral | 0.98 | 0.01 |  | 950 | 81 |  | 0.10 | 0.10 |  | 0.57 | 0.11 |
| 25 | gambling | 0.99 | 0.02 |  | 968 | 63 |  | 0.10 | 0.07 |  | 0.49 | 0.09 |
| 33 | neutral | 0.97 | 0.02 |  | 1003 | 61 |  | 0.13 | 0.11 |  | 0.41 | 0.11 |
| 33 | gambling | 0.98 | 0.02 |  | 999 | 56 |  | 0.12 | 0.09 |  | 0.39 | 0.09 |

**Table S2.** Overview of the stop-signal task pretests data. Stimuli Categorization accuracy [*p*(correct)], go response RT (in ms), probability of a missed go response [*p*(miss)] and probability of responding on stop-signal trials [*p*(respond|signal)] as a function of stimulus type and level of stop-signal probability (0%–33%). M = mean; sd = standard deviation.

**Table S3.** Overview of performance for the stop-signal task (SST). Go-signal RT (in ms), probability of a missed go response [*p*(miss)] and probability of responding on stop-signal trials [*p*(respond|signal)] as a function of level of stop-signal probability (0%–33%), SST block (one, two) and group. 25^th^,75^th^ = 25^th^ and 75^th^ precentile, sd = standard deviation.

| Group | Level of *p*(stop-signal) | Stimulus type | *p*(correct) | |  | Go RT | |  | *p*(miss) | |  | *p*(respond|signal) | |
| --- | --- | --- | --- | --- | --- | --- | --- | --- | --- | --- | --- | --- | --- |
|  |  |  | M | sd |  | M | sd |  | M | sd |  | M | sd |
| Controls | 0 | neutral | 0.98 | 0.02 |  | 594 | 85 |  | 0.02 | 0.02 |  | / | / |
|  | 0 | poker | 0.97 | 0.02 |  | 628 | 93 |  | 0.01 | 0.01 |  | / | / |
|  | 17 | neutral | 0.98 | 0.01 |  | 887 | 120 |  | 0.08 | 0.07 |  | 0.70 | 0.09 |
|  | 17 | poker | 0.99 | 0.02 |  | 896 | 118 |  | 0.05 | 0.05 |  | 0.62 | 0.19 |
|  | 25 | neutral | 0.98 | 0.01 |  | 954 | 83 |  | 0.11 | 0.08 |  | 0.54 | 0.09 |
|  | 25 | poker | 0.99 | 0.02 |  | 944 | 79 |  | 0.13 | 0.07 |  | 0.59 | 0.15 |
|  | 33 | neutral | 0.97 | 0.05 |  | 1020 | 63 |  | 0.17 | 0.14 |  | 0.36 | 0.08 |
|  | 33 | poker | 0.97 | 0.03 |  | 1013 | 85 |  | 0.19 | 0.11 |  | 0.40 | 0.13 |
| Poker players | 0 | neutral | 0.97 | 0.03 |  | 592 | 70 |  | 0.02 | 0.01 |  | / | / |
|  | 0 | poker | 0.98 | 0.02 |  | 566 | 84 |  | 0.01 | 0.01 |  | / | / |
|  | 17 | neutral | 0.98 | 0.01 |  | 840 | 116 |  | 0.07 | 0.06 |  | 0.76 | 0.16 |
|  | 17 | poker | 0.98 | 0.02 |  | 841 | 132 |  | 0.06 | 0.07 |  | 0.68 | 0.13 |
|  | 25 | neutral | 0.99 | 0.01 |  | 885 | 127 |  | 0.08 | 0.08 |  | 0.53 | 0.16 |
|  | 25 | poker | 0.98 | 0.02 |  | 883 | 100 |  | 0.10 | 0.08 |  | 0.61 | 0.20 |
|  | 33 | neutral | 0.98 | 0.02 |  | 932 | 136 |  | 0.12 | 0.11 |  | 0.49 | 0.19 |
|  | 33 | poker | 0.99 | 0.02 |  | 916 | 143 |  | 0.13 | 0.11 |  | 0.43 | 0.14 |

**Table S4.** Mean RT on go-trials and mean SSD (in milliseconds) on stop-trials for each consecutive block of trials, separately for each stop-signal probability context (green = 0%, yellow = 17%, orange = 25%, red = 33%). These values were averaged across the whole sample of participants (N = 28).

| Context | Block of Trials | *Mean* Go-signal RT (*SD*) | *Mean* SSD (*SD*) |
| --- | --- | --- | --- |
| Green | Trial 1 to 9 | 586(97) | / |
| Green | Trial 55 to 63 | 599(89) | / |
| Green | Trial 73 to 90 | 560(79) | / |
| Green | Trial 109 to 117 | 592(93) | / |
| Green | Trial 127 to 135 | 547(87) | / |
| Green | Trial 145 to 162 | 547(78) | / |
| Green | Trial 181 to 189 | 526(107) | / |
| Green | Trial 253 to 270 | 536(92) | / |
| Green | Trial 424 to 432 | 531(126) | / |
| Yellow | Trial 10 to 18 (2 stop-signals) | 712(122) | 800(0) |
| Yellow | Trial 64 to 72 (1 stop-signal) | 828(172) | 751(71) |
| Yellow | Trial 172 to 180 (1 stop-signal) | 855(198) | 719(107) |
| Yellow | Trial 190 to 198 (3 stop-signals) | 900(179) | 740(108) |
| Yellow | Trial 280 to 288 (3 stop-signals) | 876(221) | 747(167) |
| Yellow | Trial 307 to 315 (1 stop-signal) | 862(217) | 757(175) |
| Yellow | Trial 325 to 333 (1 stop-signal) | 875(178) | 749(179) |
| Yellow | Trial 361 to 387 (4 stop-signals) | 883(142) | 692(206) |
| Yellow | Trial 406 to 423 (2 stop-signals) | 898(155) | 725(178) |
| Orange | Trial 19 to 27 (3 stop-signals) | 902(188) | 773(23) |
| Orange | Trial 37 to 54 (5 stop-signals) | 932(92) | 763(51) |
| Orange | Trial 136 to 144 (2 stop-signals) | 888(157) | 768(92) |
| Orange | Trial 163 to 171 (2 stop-signals) | 948(162) | 777(111) |
| Orange | Trial 208 to 234 (5 stop-signals) | 949(125) | 725(116) |
| Orange | Trial 271 to 279 (2 stop-signals) | 841(176) | 724(140) |
| Orange | Trial 316 to 324 (4 stop-signals) | 896(151) | 716(174) |
| Orange | Trial 343 to 351 (1 stop-signal) | 877(168) | 743(172) |
| Orange | Trial 388 to 396 (4 stop-signals) | 887(158) | 703(184) |
| Red | Trial 28 to 36 (4 stop-signals) | 1013(113) | 769(23) |
| Red | Trial 91 to 108 (7 stop-signals) | 944(163) | 767(76) |
| Red | Trial 118 to 126 (1 stop-signal) | 932(178) | 740(100) |
| Red | Trial 199 to 207 (4 stop-signals) | 1015(145) | 737(122) |
| Red | Trial 235 to 252 (5 stop-signals) | 944(154) | 730(134) |
| Red | Trial 289 to 306 (6 stop-signals) | 968(159) | 723(150) |
| Red | Trial 344 to 342 (2 stop-signals) | 919(145) | 741(160) |
| Red | Trial 352 to 360 (3 stop-signals) | 975(214) | 720(177) |
| Red | Trial 397 to 405 (4 stop-signals) | 951(156) | 707(181) |

**Table S5. Significant activations within the whole sample of participants (*N* = 28) for proactive motor response inhibition**

|  |  | **MNI**  **Coordinates** | | | | | |
| --- | --- | --- | --- | --- | --- | --- | --- |
|  | **Structure** | **Left/**  **right** | **k** | ***x*** | ***y*** | ***z*** | ***Z***  ***value*** |
| Parametric effect of stop-signal probability on go-signal activation | Occipital Pole, Insula, Frontal Operculum Cortex, Frontal Pole, Frontal Orbital Cortex, Lingual Gyrus, Inferior Frontal Gyrus, extending into Caudates and Putamen | L/R | 23757 | -6 | -98 | -8 | 5.57 |
|  |  |  |  | -30 | 24 | 6 | 5.37 |
|  |  |  |  | 36 | 24 | -4 | 5.24 |
|  |  |  |  | -42 | 20 | 6 | 5.23 |
|  |  |  |  | 46 | 18 | -2 | 5.22 |
|  |  |  |  | 14 | -94 | 0 | 5.21 |
|  | Supramarginal Gyrus, Inferior Parietal Lobule, Middle Temporal Lobe, Superior Temporal Gyrus | R | 2809 | 56 | -40 | 38 | 4.57 |
|  |  |  |  | 58 | -44 | 44 | 4.54 |
|  |  |  |  | 48 | -36 | 50 | 4.47 |
|  |  |  |  | 62 | -52 | 14 | 4.40 |
|  |  |  |  | 52 | -48 | 46 | 4.40 |
|  |  |  |  | 54 | -54 | 38 | 4.21 |
|  | Superior Temporal Gyrus, Postcentral Gyrus, Inferior Parietal Lobule | L | 2768 | -64 | -28 | 16 | 4.63 |
|  |  |  |  | -52 | -20 | 28 | 4.59 |
|  |  |  |  | -60 | -22 | 26 | 4.58 |
|  |  |  |  | -64 | -16 | 24 | 4.51 |
|  |  |  |  | -62 | -42 | 16 | 4.46 |
|  |  |  |  | -62 | -38 | 16 | 4.39 |
|  | Precuneus Cortex, Lateral Occipital Cortex | R | 535 | 14 | -66 | 46 | 3.91 |
|  |  |  |  | 4 | -66 | 58 | 3.86 |
|  |  |  |  | 8 | -62 | 56 | 3.81 |
|  |  |  |  | 14 | -70 | 62 | 3.80 |
|  |  |  |  | 4 | -56 | 58 | 3.69 |
|  |  |  |  | 10 | -56 | 56 | 3.65 |
|  | Cingulate Gyrus | L/R | 152 | 2 | -20 | 28 | 4.14 |
|  | Postcentral Gyrus | R | 115 | 56 | -16 | 28 | 3.84 |

k, voxel cluster size (each voxel = 8mm^3^); L, left; R, right; MNI, Montreal Neurological Institute.

**Table S6. Significant activations within the whole sample of participants for reactive motor response inhibition.**

|  |  |  |  | | **MNI**  **Coordinates** | | | | | | |  |  |
| --- | --- | --- | --- | --- | --- | --- | --- | --- | --- | --- | --- | --- | --- |
|  | | **Structure** | | **Left/**  **right** | | **BA** | **k** | ***x*** | ***y*** | ***z*** | ***Z***  ***value*** | | |
| Successful stop minus unsuccessful stop | | Frontal Orbital Cortex | | L | | - | 571 | -22 | 40 | -16 | 3.91 | | |
|  | | Occipital Pole | | R | | - | 538 | 22 | -98 | -2 | 4.00 | | |
|  | | Putamen | | R | | - | 474 | 18 | 10 | -8 | 4.22 | | |
|  | | Middle Frontal Gyrus | | R | | - | 436 | 30 | -4 | 54 | 4.32 | | |
|  | | Lateral Occipital Cortex | | R | | 7 | 377 | 24 | -62 | 52 | 4.03 | | |
|  | | Occipital Pole | | L | | - | 311 | -20 | -98 | -4 | 3.73 | | |
|  | | Putamen | | R | | - | 304 | -16 | 8 | -8 | 4.32 | | |
|  | | Putamen | | L | | - | 283 | -22 | 4 | 8 | 4.16 | | |
|  | | Anterior Cingulate | | L | | 32 | 226 | -4 | 48 | -4 | 3.95 | | |
|  | | Superior Frontal Gyrus | | L | | 10 | 201 | -20 | 66 | 2 | 4.16 | | |
|  | | Superior Frontal Gyrus | | R | | 9 | 144 | 28 | 38 | 26 | 3.75 | | |
|  | | Middle Frontal Gyrus | | L | | 6 | 126 | -24 | -6 | 48 | 3.83 | | |
|  | | Cingulate Gyrus | | L | | - | 124 | -4 | -34 | 32 | 3.88 | | |
|  | | Lateral Occipital Cortex | | R | | - | 114 | 50 | -74 | 2 | 3.80 | | |
|  | | Superior Frontal Gyrus | | L | | 6 | 110 | -22 | 24 | 56 | 3.88 | | |
|  | | Medial Frontal Gyrus | | L/R | | - | 96 | 0 | 50 | -18 | 3.49 | | |

**Table S7. Higher brain activations in poker players, as compared to controls, for the successfull stop minus baseline contrast.**

|  |  |  |  | | **MNI**  **Coordinates** | | | | | | |  |
| --- | --- | --- | --- | --- | --- | --- | --- | --- | --- | --- | --- | --- |
|  | | **Structure** | | **Left/**  **right** | | **BA** | **k** | ***x*** | ***y*** | ***z*** |  |  |
|  | | Cerebellum, Temporal Occipital Fusiform Cortex | | R | | - | 27387 | 36 | -56 | -24 |  |  |
|  | | Amygdala | | R | | - | 486 | 22 | -4 | -28 |  |  |
|  | | Middle Temporal Gyrus | | L | | - | 186 | -52 | -14 | -20 |  |  |
|  | | Paracingulate Gyrus | | R | | 32 | 104 | 12 | 20 | 32 |  |  |
|  | | Caudate | | L | | - | 70 | -12 | 20 | 16 |  |  |
|  | | Precentral Gyrus, Inferior Frontal Gyrus | | R | | 9 | 37 | 60 | 8 | 24 |  |  |

Between-groups differences were tested using non-parametric permutation methods for inference on statistic maps, with 10,000 random permutations of the data. Maps resulting from between-groups analyses were thresholded using Threshold-free cluster enhancement (TFCE) with *p* < .05 corrected for multiple comparisons across the whole brain. k, voxel cluster size (each voxel = 8mm^3^); L, left; R, right; MNI, Montreal Neurological Institute.

**Table S8. Significant activations within the whole sample of participants for poker cue reactivity during proactive and reactive response inhibition.**

|  |  |  |  | | **MNI**  **Coordinates** | | | | | | |  |  |
| --- | --- | --- | --- | --- | --- | --- | --- | --- | --- | --- | --- | --- | --- |
|  | | **Structure** | | **Left/**  **right** | | **BA** | **k** | ***x*** | ***y*** | ***z*** | ***Z***  ***value*** | | |
| Poker cue (parametric effect of stop-signal probability on go-signal activation) minus neutral cue (parametric effect of stop-signal probability on go-signal activation) | | Precentral Gyrus, Postcentral Gyrus | | L | | 3 | 749 | -40 | -20 | 52 | 4.14 | | |
| Poker cue (successful stop) minus neutral cue (successful stop) | | Occipital Pole | | L/R | | 17 | 5696 | -10 | -98 | -4 | 5.10 | | |
| Poker cue (unsuccessful stop) minus neutral cue (unsuccessful stop) | | Occipital Pole | | L/R | | 17 | 4568 | -12 | -98 | -4 | 4.96 | | |
|  | | Precentral Gyrus, Postcentral Gyrus | | L | | 4 | 1847 | -34 | -24 | 62 | 4.84 | | |
